# Effects of the Individual and Pair Housing of Calves on Long-Term Heifer Production on a UK Commercial Dairy Farm

**DOI:** 10.3390/ani14010125

**Published:** 2023-12-29

**Authors:** Sophie A. Mahendran, D. Claire Wathes, Richard E. Booth, Nicola Blackie

**Affiliations:** Department of Pathobiology and Population Sciences, Royal Veterinary College, Hawkshead Lane, Hertfordshire AL9 7TA, UK

**Keywords:** calf housing, pair, individual, production, fertility, culling, long term

## Abstract

**Simple Summary:**

The short-term benefits of pair housing pre-weaning calves have been successfully demonstrated, but the longer-term implications are still relatively unknown, especially for heifers enter the milking herd. This study aimed to investigate the impact of pairs compared to the individual housing of pre-weaning calves on longer-term heifer production. We found that pair-housed calves were less likely to exit the herd, which resulted in pair-housed calves producing more total milk per calf recruited into the original study. However, pair-housed calves also suffered from higher risks of developing udder health issues. The season of birth had an impact on growth rates, with heifers born in the colder winter months having reduced growth, but seasonality did not impact any other variables.

**Abstract:**

Pair housing of pre-weaning dairy calves has previously demonstrated positive impacts on their growth, health and behaviour, but longer-term effects on production are still relatively unknown. This study followed a cohort of 431 Holstein heifers, recruited from a single UK commercial dairy farm, from weaning until either culling or the end of their first lactation. All animals were allocated to either individual or pair housing as a pre-weaning calf. Following weaning, all heifers were similarly managed through group housing, feeding with total mixed rations, the use of automatic heat detection for artificial insemination and weighing every two months until conception. Farm staff identified disease occurrences, which were treated following standard operating procedures. First-lactation monthly milk recording was used to measure milk yields and somatic cell counts. Overall mortality (voluntary and involuntary) was 26.6%, with a decreased hazard of exiting the herd if the heifer was pair housed as a calf (HR 0.70; *p* = 0.067). The voluntary cull rate was highest in the post-insemination period (13.0%) due to poor fertility. Heifers that were pair housed as calves had significantly increased odds of developing udder health issues as a primiparous cow (OR = 1.93, *p* = 0.022). Despite this, the 305-day milk yields were not associated with the housing group. However, the total milk produced per calf recruited into the original study was greater for pair-housed compared with individually housed calves (8088 kg vs. 7115 kg; *p* = 0.071), which is likely due to the significantly higher hazard of individually housed calves exiting the herd prematurely.

## 1. Introduction

The short-term importance of social pairing compared to individual housing for pre-weaning calves has been well studied; however, direct comparisons of the longer-term impacts of these housing types is currently lacking. Pair-housed calves have a shorter latency before starting to consume solid feeds [1], visit and spend more time at the concentrate feeder [1], consume more starter [2,3,4] and have higher weight gains post-weaning [1,2] compared with individually housed calves. However, it is unclear if these advantages continue throughout the rest of the rearing period and into lactation.

Growth rates of heifers is a key performance indicator, with targets of 0.7–0.8 kg/day needed to achieve an age at first calving of 24 months [5] and an optimum pre-partum bodyweight of 595–645 kg for Holstein heifers [6]. Slower-growing young calves (such as those individually housed) can exhibit some compensatory skeletal growth, but there may be alterations in the proportions of bone, muscle, fat and viscera, resulting in heifers failing to achieve their genetic potential [7]. Adequate growth is also vital for the prompt attainment of puberty and regular oestrous cycles that allow heifers to conceive and, therefore, enter the milking herd following parturition with an age at first calving of around 2 years [8]. In addition to fertility, growth rates are strongly linked to milk production. MacDonald et al. [9] demonstrated that higher growth rates during the post-weaning period lead to a 7% milk-yield increase, and for every 1 kg/day increase (or part thereof) in early-life daily liveweight gain (DLWG), heifers can produce between 1003 kg [10] and 1113 kg [11] more milk in the first lactation. Similarly, Hayes et al. [12] found that heifers growing at 0.82 kg/day would produce a 1120 kg greater 305-day standardised milk yield than those growing at only 0.55 kg/day, and 218 kg more than a heifer growing at 0.7 kg/day. Given the links of housing type with early-life growth rates, it is important to establish if the choice of individual compared to pair housing of calves impacts their reproductive performance around puberty and potential milk production as primiparous cows.

Disease prevalence in calves can be affected by housing type, such that individually housed calves either experience higher disease levels [13,14,15] or no difference in disease prevalence compared to individually housed calves [16]. Calf disease can reduce growth rates during the rearing period [17], especially up to 6 months of age [18], possibly due to disease immune responses creating a large bio-energetic challenge [19] as well as potentially reducing feed intakes [20]. Specific diseases, such as bovine respiratory disease, have demonstrated negative impacts on reaching first insemination or achieving first calving, whereas calf diarrhoea was associated with heifers requiring more inseminations to become pregnant and a 325 kg reduction in first-lactation yields [21]. Other studies have not demonstrated long-term negative impacts of calf disease on survival [22] or first-lactation milk production [23], with lung consolidation at weaning not being linked to the weekly average milk production [24].

Further adult production parameters that could be impacted by calf housing type are udder damage, mastitis, and milk loss in the lactating animal due to the occurrence of intersucking [25]. This is an abnormal behaviour of group-housed cattle following weaning [26] and occurs when one heifer sucks on the udder of a herdmate [27]. This can lead to teat damage and mastitis through the opening of the teat canal, allowing bacteria to enter the udder [28]. The prevalence of intersucking has been reported at between 1.1–1.4% of UK farms affected [29], but this is much lower than in other countries, who report between 26.3% [30] and 100% [25,31] of farms having cattle seen to be exhibiting this behaviour. The within-farm prevalence is reported to be lower, with between 0.5–15% of cows within a herd noted to carry out this behaviour [32]. Prevention strategies include the use of pronged nose plates or rings which provoke avoidance behaviours in the cattle which are to be sucked, but these methods raise welfare concerns [33]. Development of intersucking in adult cattle may be linked to habit formation via cross-sucking behaviour in pair-housed calves [34], especially around the transition from milk to solid feed [30,35].

All of the production parameters mentioned (growth rates, disease prevalence, udder health issues and milk yield) can impact the culling of dairy cattle, which is the removal of animals from the herd voluntarily due to sale or slaughter or involuntarily due to salvage or death [36]. Cull rates for a herd will determine the number of replacement heifers needed to maintain a herd size, with many farms now reporting herd turnover levels of 35–40% [37]. However, poor survivability of replacement heifers will reduce their availability, meaning that farms are less able to voluntarily cull mature animals with poor fertility or health or low milk production. This can have negative economic consequences for a farming enterprise, with the prolonged retention of economically inferior cows. There is also a cost attributable to rearing each heifer that does not reach lactation, which ranges from GBP 103.49 to GBP 146.19/surviving heifer [38]. Understanding calf management decisions that can impact the culling rates of the heifers produced can therefore have positive consequences for herd replacement rates and economics. One of these decisions is the housing strategies used for calves.

Another positive aspect linked to the social housing of calves is their behavioural development, with the provision of more complex social environments for young animals potentially improving their capacity to cope with novel environments encountered at regrouping [39]. Social relationships are also most strongly formed before 3.5 months of age, as demonstrated by companion choice and lying-proximity patterns. Calves that have been together from two weeks of age develop preferential relationships that can last for more than 1.5 years [40]. This may be helpful to heifers as they move through the production cycle of their first insemination, calving and lactation, where environment and group changes often occur at a higher frequency. In addition, calves reared in individual housing demonstrate increased fear responses [41], with adult cows that fear people having 70% more residual milk that is unable to be harvested at milking due to the inhibition of oxytocin release [42]. This suggests that the negative impacts imposed by individual housing may go on to affect production parameters as an adult.

The aim of this study was to investigate the impact of pair compared to individual housing of pre-weaning calves on longer-term heifer production, which included measuring growth rates, health, fertility and first-lactation milk yields.

## 2. Materials and Methods

This study was approved by the Clinical Research Ethics Committee of the Royal Veterinary College (protocol code: URN SR2019-0369, 27 March 2020).

### 2.1. Calf Enrolment and Management

The study was conducted on a single commercial dairy farm in the southwest of England that milks 1800 Holstein and Jersey dairy cows in an all-year-round calving pattern. A total of 431 of the Holstein heifer calves were recruited at birth from March to December 2020. Details of the pre-weaning management are given in Mahendran et al. [13]; in summary, calves were systematically allocated to either individual or pair housing, which consisted of pens within sheds formed from prefabricated plastic dividers (Calf-Tel Pen system, Hampel Corp, Germantown, WI, USA), with internal dimensions of 122 cm × 213 cm for an individual pen and twice this for a pair pen. Calves were fed 6L of milk replacer per day (24% whey protein, 20% fat), ad libitum starter pellets (18% crude protein, 4% fats, 12% crude fibre) and had ad libitum water access. Additional fibre was provided as straw bedding, which was replenished daily. Pre-weaned calves received an additional total mixed ration (TMR) from 4 weeks of age (Table 1). Calves were step-weaned off milk between 7–8 weeks of age, with calves moved out of their pre-weaning housing at approximately 9 weeks of age.

During the pre-weaning period, calves received weekly visits from the researcher (SM), during which calves underwent a clinical health assessment following a modified Wisconsin scoring system [43,44] to assess their demeanour, nasal and ocular discharge, cough, faecal consistency, rectal temperature, navel and joint health. A diagnosis of bovine respiratory disease was given when a calf displayed at least one sign of upper respiratory disease (nasal/ocular discharge or cough) and pyrexia (≥39.5 °C).

### 2.2. Post-Weaning Management, Breeding and Nutrition

Following weaning, calves were loose-housed in groups of 30 in straw-bedded pens at the calf unit, grouped by age. Each calf was weighed using electronic weigh scales (Bateman, Cheddleton, UK) approximately every 2 months by farm staff up until the heifers were confirmed as pregnant. Calves continued to be fed the same TMR ration (Table 1). These groups remained stable until approximately 6 months of age when they were moved to a separate heifer fertility site.

At the fertility site, heifers continued to be group-housed in straw-loose yards and were fed a different TMR ration (Table 1). At the fertility site, a commercially available pedometer system with automatic oestrus detection was placed onto the forelimb of each heifer and used to identify those eligible for insemination (Genus ABS Breeder Tag System, DeForest, WI, USA). Heifers were served by staff from a commercial fertility company using artificial insemination, with inseminations reported to commence upon the Holstein heifers reaching approximately 400 kg as measured by routine weighing. Heifers were given up to three straws of sexed semen, followed by enrolment into a synchronisation program using follicle-stimulating hormones (FSH), luteinising hormone (LH) and conventional semen strawsfor up to a total of eight inseminations. Pregnancy diagnosis was carried out by the routine veterinary practice between 30–50 days post-service.

Approximately 21 days pre-calving, heifers were moved to the transition cow facility at the main dairy and housed in a loose-straw yard in a heifer-only group prior to calving. They were fed a DCAB diet at 313 mEq/kg. Following calving, these first-lactation heifers were kept as a single group, housed in deep-bedded sand cubicles and milked three times per day. They were fed the TMR ration for maintenance plus an additional 45 L (Table 1).

Following weaning, all daily health checks were carried out by multiple farm staff using standard operating procedures (SOPs) developed by the farm’s veterinary practice. Staff were trained by the veterinary practice to recognise and record disease incidents, including respiratory disease (defined as a temperature ≥ 39.0 °C and either a cough, mucopurulent nasal discharge or tachypnoea), lameness (defined as a mobility score of 2 or 3 [45]), clinical mastitis (defined as milk changes along with heat or swelling of the udder) and subclinical mastitis (defined as a somatic cell count ≥200,000 cells/mL with no clinical signs of mastitis). Lame heifers had their feet examined by a trained foot trimmer on the farm. All diseases were treated according to farm SOPs. Disease events were recorded on the farm software (Uniform-Agri, Assen, The Netherlands), from which they were extracted for data analysis. Episodes of intersucking (defined as one primiparous cow sucking on the udder of another) observed by staff whilst in the lactating herd were recorded by staff, along with the occurrence of quarter loss in primiparous cows. Mortality events for voluntary and involuntary culls were also recorded from farm records.

Whilst in the lactating herd, primiparous cows underwent monthly milk recording conducted by a commercial company (NMR, UK). This measured the volume and solid components of the milk, along with the somatic cell count (SCC). A standardised energy-corrected 4.0% fat and 3.3% protein milk yield (ECM) was calculated for the estimated milk volume given for up to 305 days of milk [46]:

ECM (kg) = milk (L) × ((0.383 × fat (%) + 0.242 × protein (%) + 0.7832)/3.1138)

### 2.3. Calves Born to Recruited Heifers

Data on the morbidity and mortality rates in the calves born to the recruited heifers at their first calving were collected from their birth until weaning at eight weeks of age. Dams calved in individual straw pens. The dam was milked within one hour of calving, and two 3 L feeds of colostrum were given six hours apart to her calf via an oesophageal tube feeder. The calf was then transported to the calf-rearing unit and placed into shed housing containing prefabricated pens (the same as the original pair housing used in this study), with all animals kept in pairs. The calves remained in these pairs until post-weaning. The calves’ feed and management was the same as previously described for the recruited heifers. Farm records were used to collect data on the mortality and disease occurrence in these offspring up until eight weeks of age.

### 2.4. Statistical Analysis

All data were stored in Excel (Microsoft Office; Microsoft, Redmond, WA, USA). All analyses were performed using SPSS (Version 27.0, IBM SPSS Statistics for Windows, IBM Corp, New York, NY, USA). Significance was declared at *p* ≤ 0.05, and trends were reported if *p* ≤ 0.10. For all analyses, the assumption of normality was assessed through the visual inspection of plots. A sample-size calculation was based on identifying a 500 L milk-yield difference in the 305-day milk yield of heifers at the end of their first lactation, taking into account that approximately 30% of born heifers do not survive to the end of their first lactation [47]. Using a 2-tailed test, a variance of 0.10, a confidence level of 0.95 and a power of 0.8, the sample size for detecting a significant difference was 150 individually housed and 300 pair-housed calves (150 pairs of calves).

A total of 431 calves were initially recruited at birth into the study over a nine-month period, with 13 calves dying during the pre-weaning period and being excluded from further analysis (a 3.0% pre-weaning mortality rate). Full analysis of the pre-weaning data is presented in the paper by Mahendran et al. [13].

A summary of the statistical analyses, with the specific dependent and independent variables utilised, are given in Appendix A. Following an initial descriptive analysis of disease, events were combined to create a binary disease variable whereby 1 indicated heifers that had experienced any form of disease, and 0 indicated a heifer that had never experienced any form of disease. Season of birth was classified into Spring (April and May), Summer (June, July and August), Autumn (September and October) and Winter (January, February and March). Cox regression for survival analysis was used to evaluate the risk of exiting the herd between weaning and the end of first lactation (both involuntary deaths and voluntary culls).

Binary logistic generalised estimating equations with a logit link function and an exchangeable working matrix was used to assess the outcomes of binary disease occurrence from weaning to the end of first lactation (excluding udder health issues), udder health issues (including mastitis, high somatic cell count (≥200,000 cells/mL) and losing a quarter), achieving a successful pregnancy and reaching parturition in all recruited cattle. The mortality rate and the disease occurrence up to the weaning of calves born to the recruited heifers at their first calving were also assessed. The variable ”pen” was used to account for clustered measures within a pair of calves. The outcome of the mean daily liveweight gain (DLWG) from birth up to the confirmation of pregnancy, the number of inseminations given, the age at first calving, and the energy-corrected first-lactation 305-day milk yield for the Holstein heifers were analysed using linear mixed-effects models. Pen and calf identification number were included as random effects in each model, and results were reported as F-values in the format F_(numerator df, denominator df)_.

Additional data were collected on heifer milk production for those animals that did not reach a 305-day lactation length. This included heifers that exited the herd due to death or culling during lactation and heifers that dried off early. Heifers that exited prior to parturition were also included; these were listed with a milk yield of 0 kg. These data were used to calculate the total amount of milk produced per heifer originally recruited into each housing group, which was then compared across the housing groups via the Mann–Whitney U test.

## 3. Results

### 3.1. Daily Liveweight Gain

**Figure 1:**
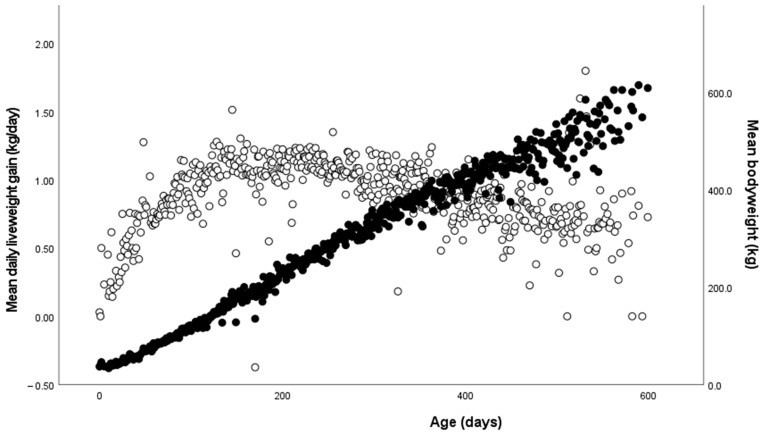
Age (in days) of the heifers plotted against the mean daily liveweight gain on the left axis (white circles) and bodyweight on the right axis (black circles). The bodyweight has a linear association with age, with an R^2^ = 0.95. There was an overall trend of an increasing DLWG until approximately 200 days of age, followed by a decrease until heifers were confirmed as pregnant.

The mean (±SD) daily liveweight gain (DLWG) from birth to the confirmation of pregnancy for Holstein heifers was 0.73 ± 0.12 kg/day (Figure 1). Birthweight of the calf showed a significant association with the DLWG (F_1,386_ = 84.60, *p <* 0.001), such that a 1 kg increase in birthweight led to a 0.012 kg/day increase (R^2^ = 0.18). There was a significant association with season of birth (F_3,246_ = 3.56, *p =* 0.015); calves born in the winter season had a lower DLWG than in the other seasons (Winter, 0.67 kg/day; Spring, 0.75 kg/day; Summer, 0.72 kg/day; Autumn, 0.75 kg/day). Occurrence of pre-weaning disease was also significantly associated (F_1,406_ = 20.2, *p* < 0.001), with those suffering from disease pre-weaning growing at 0.70 (SE 0.010) kg/day compared with healthy calves growing at 0.74 (SE 0.008) kg/day. However, the presence of disease from weaning to first insemination was not associated with the DLWG (F_1,400_ = 0.047, *p =* 0.83). There was a trend with the housing group (F_1,296_ = 2.90; *p =* 0.09), such that individually housed calves had a higher DLWG (0.73 kg/day compared with 0.71 kg/day).

### 3.2. Mortality and Morbidity

**Figure 2:**
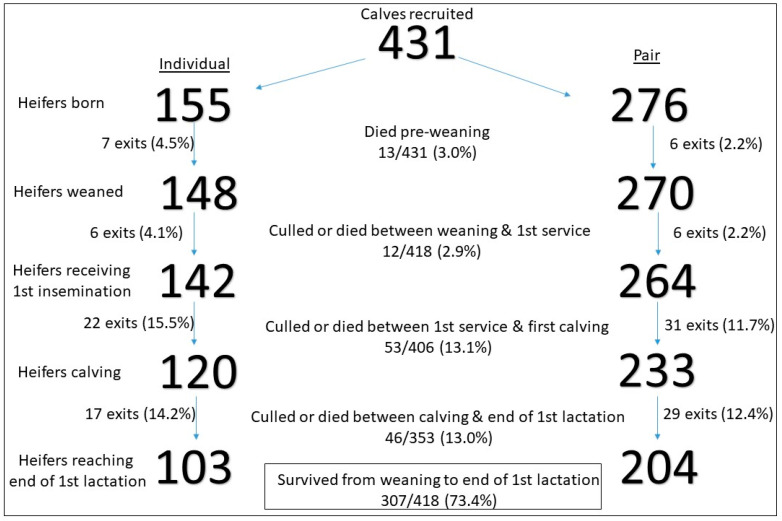
Summary of the total number of calves retained and exiting (combined culled and died) from the study.

A total of 11/418 (2.6%) of heifers were involuntarily culled between weaning and the end of first lactation (5 pre-insemination, 2 following insemination but prior to first calving, and 4 in first lactation). A further 101/418 (24.2%) of heifers were voluntarily culled (Figure 2). The predominant reason for voluntary culling between the first insemination and calving was poor fertility (51/101; 50.5%). Between calving and the end of first lactation, the main reasons were injuries (20/101; 19.8%), udder-related problems (8/101; 7.9%) and lameness (8/101; 7.9%).

**Figure 3:**
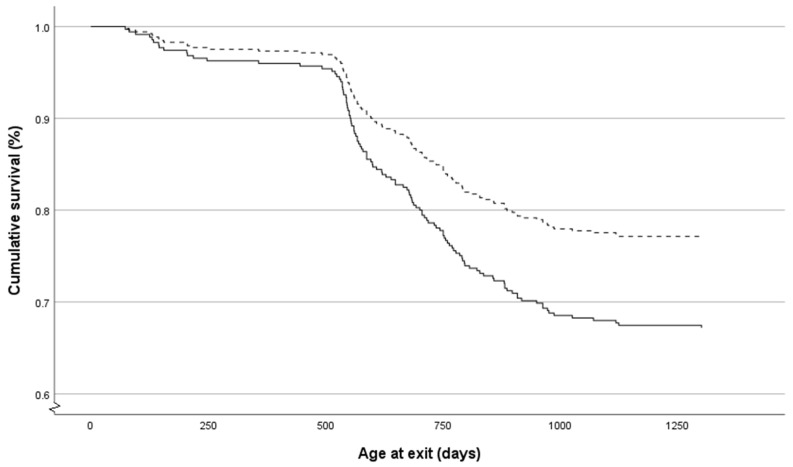
Cox regression survival plot for heifers housed individually pre-weaning (solid line) compared to those housed in pairs (dashed line). Individually housed calves had an increased hazard of exiting the herd by the end of first lactation (*p* = 0.067).

Cox regression analysis for the risk of exiting the herd between weaning and the end of first lactation demonstrated a trend for association with the pre-weaning housing group; pair-housed calves had a lower hazard of exiting (HR 0.70; 95% CI 0.48–1.03; *p* = 0.067) (Figure 3). There was no significant association with pre-weaning binary disease occurrence (HR 0.92; 95% CI 0.62–1.37; *p* = 0.92) or post-weaning binary disease occurrence (HR 1.12; 95% CI 0.79–1.77; *p* = 0.41) and the risk of exiting the herd.

### 3.3. Disease

Out of all the calves enrolled, a total of 141/431 (32.7%) experienced disease between birth and weaning [13]. Of the heifers surviving to weaning, a further 273/418 (65.3%) experienced at least one case of disease between weaning and the end of the first lactation (134/418 prior to the first insemination and 139/418 between the first insemination and the end of the first lactation) (Table 2). Out of all the heifers experiencing disease, 106/273 (38.8%) animals suffered multiple different disease events, with the most common combination involving lameness alongside another disease.

The model assessing associations with disease occurrence from weaning to end of first lactation (excluding udder health issues of mastitis and high SSC, which were analysed separately) found that none of the following variables were significantly associated: season of birth (*p* = 0.12); housing group (OR = 0.95; *p* = 0.84); pre-weaning disease occurrence (OR =1.13; *p* = 0.58); mean DLWG up until conception (OR = 0.87; *p* = 0.87).

### 3.4. Fertility

A total of 406/431 heifers survived to puberty and received a first insemination (Table 3). Assessment of the number of inseminations given to heifers demonstrated no associations with any of the following variables: housing group (F_1,287_ = 0.50; *p =* 0.48); season of birth (F_3,220_ = 1.60; *p =* 0.19); occurrence of disease pre-weaning (F_1,361_ = 0.31; *p =* 0.58) or disease between weaning and first insemination (F_1,359_ = 0.089; *p* = 0.77); DLWG from birth to first insemination (F_1,361_ = 0.011; *p =* 0.92); weight at first insemination (F_1,360_ = 0.29; *p =* 0.59); age at first insemination (F_1,356_ = 0.58; *p =* 0.45).

The age at first calving was not associated with the housing group (F_1,261_ = 0.85; *p =* 0.36), season of birth (F_3,310_ = 1.31; *p =* 0.27), pre-weaning disease (F_1,311_ = 2.20; *p =* 0.14), disease post-weaning to calving (F_1,311_ = 0.002; *p =* 0.97), DLWG (F_1,312_ = 0.95; *p =* 0.33), nor was there any interaction between the season of birth and the DLWG (F_3,311_ = 0.75; *p =* 0.52). There was a trend for the weight at first insemination (F_1,312_ = 2.81; *p =* 0.095), such that as the weight increased, so did the age at calving (R^2^ = 0.005).

Assessment of achieving pregnancy and reaching parturition was significantly associated with the number of inseminations a heifer received (OR = 0.47, 95% CI 0.38–0.58; *p* < 0.001) such that an increasing number of inseminations led to reduced odds of achieving a successful pregnancy. There was no association with the housing group (*p* = 0.37), season of birth (*p* = 0.38), binary pre-weaning disease (*p* = 0.48), binary disease from weaning until insemination (*p* = 0.75), age at first insemination (*p* = 0.65), neither was there any interaction between the season of birth and the DLWG (*p* = 0.59).

### 3.5. Milk Yield and Udder Health

A total of 307/418 (73.4%) heifers completed a 305-day first lactation, with a mean standardised energy-corrected milk yield of 9995.8 ± 1235.6 kg (SD). Nine heifers were noted to carry out intersucking at any point between post-weaning and the end of first lactation (three from individual housing and six from pair housing).

**Figure 4:**
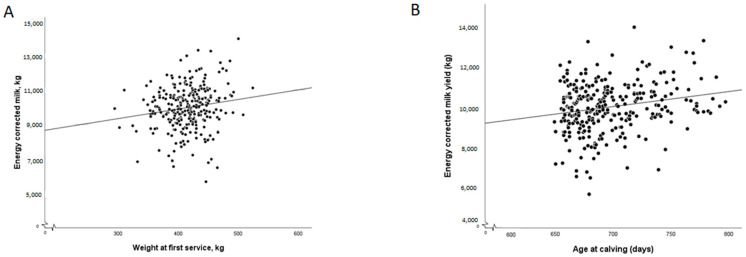
Plot (**A**) demonstrates the energy-corrected milk yield produced per heifer compared with their weight at first insemination. There is a numerical trend for an increasing milk yield as the heifer weight increases, although the effect size is low; R^2^ = 0.025. Plot (**B**) demonstrates the energy-corrected milk yield produced per heifer compared with their age at first calving. There is a numerical trend for an increasing milk yield as the age at calving increases, although the effect size is low; R^2^ = 0.043. Each dot represents one heifer.

The 305-day standardised milk-yield analysis demonstrated a significant association with the weight at first insemination (F_1,265_ = 8.83; *p =* 0.003) and with the age at calving (F_1,265_ = 9.92; *p =* 0.002) such that as both increased, so did the milk yield of the heifers (Figure 4). There was no association with the housing group (F_1,237_ = 0.074; *p =* 0.79), season of birth (F_3,263_ = 0.001; *p =* 0.99), DLWG (F_1,258_ = 0.83; *p =* 0.36), the interaction term between season of birth and DLWG (F_3,260_ = 0.11; *p =* 0.95), pre-weaning binary disease occurrence (F_1,261_ = 0.20; *p =* 0.66) or post-weaning binary disease occurrence, including udder health issues (F_1,258_ = 0.009; *p =* 0.93).

There were 83/418 (19.9%) heifers that experienced a case of clinical mastitis (Table 2), with 24 heifers losing a quarter (6/145 (4.1%) from individual housing, and 18/273 (6.6%) from pair housing). The model assessing binary occurrence of udder health issues (including mastitis, high somatic cell count and losing a quarter) indicated that this was significantly associated with the pre-weaning housing group, such that pair-housed calves had increased odds of udder health issues (OR = 2.13, 95% CI 1.16–3.92; *p* = 0.015). Overall, 19/145 (13.1%) individually housed calves and 72/273 (26.4%) pair-housed calves went on to experience udder health issues. There was no association with post-weaning binary disease occurrence, excluding udder health issues (*p* = 0.31), DLWG (*p* = 0.59), age at first calving (*p* = 0.52) or season of birth (*p* = 0.34).

**Figure 5:**
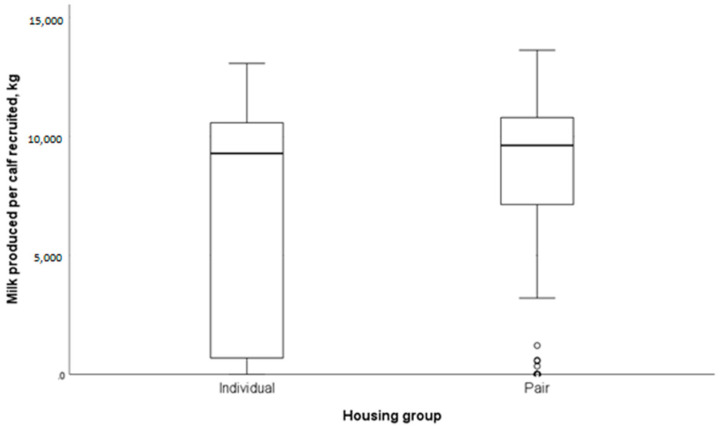
Box plots of the milk produced per heifer initially recruited into the housing study. The interquartile range for individually housed calves was 9265 kg compared with 3351 kg for pair-housed calves. Much of the variation was due to the high number of exits from the herd, which was significantly higher in individually housed compared with pair-housed calves. The circles represent outlier calves.

Assessment of the total milk produced per calf recruited into each housing group found that overall, individually housed calves had a trend towards producing less mean milk per calf recruited than pair-housed calves (mean 7194 kg vs. 8038 kg; *p* = 0.071) (Figure 5).

### 3.6. First-Calf Vitality

Out of the recruited heifers that survived to parturition, 345/353 (97.7%) gave birth to calves, with 22/345 (6.4%) dying at birth. The model for calf mortality between 24 h old and weaning indicated that 15/345 (4.3%) died, but there was no significant association with the dam’s housing group (*p* = 0.50), the dam’s DLWG (*p* = 0.93), occurrence of disease in the dam prior to calving (*p* = 0.89), the number of inseminations required to establish pregnancy (*p* = 0.29), the age at calving (*p* = 0.60) or the season in which the parturition occurred (*p* = 0.67).

Disease occurred in 206/345 (59.7%) of the calves born. The model for binary calf disease occurrence prior to weaning demonstrated there was a significant association with the season that parturition occurred in (*p* < 0.001), with calves born in spring (OR = 3.39, 95% CI 1.84–6.26; *p* < 0.001) and summer (OR = 2.38, 95% CI 1.32–4.29; *p* = 0.004) having a higher odds of disease compared with those born in the winter, but there was no difference compared to those born in autumn (OR = 1.91, 95% CI 0.48–2.92; *p* = 0.70). There was no significant association with the dam’s DLWG (*p* = 0.61), the dam’s housing group (*p* = 0.98), occurrence of disease in the dam prior to calving (*p* = 0.14), the age of the dam at calving (*p* = 0.47) or the number of services required to establish pregnancy (*p* = 0.51).

## 4. Discussion

This study followed a cohort of heifers that were housed in either individual or pair pens as pre-weaning calves and assessed the impacts that this had on their subsequent growth, health, fertility and milk yield parameters up until the end of first lactation. It was based on a single commercial dairy farm, which was managed under the guidelines stipulated by two farm assurance schemes that are used widely in the UK (Red Tractor, https://redtractor.org.uk/ and Arla, https://news.arlafoods.co.uk/sustainable, both accessed on 30 May 2023), which cover around 95% and 27% of UK dairy farms, respectively. The single-farm setting ensured that all heifers were managed under the same conditions, with the exception of the pre-weaning housing group. However, the management of replacement heifers can vary widely between farming enterprises, with heifers in this study having no access to grazing. This may mean that findings on extensively managed units could vary with those reported here.

### 4.1. Mortality

Overall, there was a trend for a decreased hazard of exiting the herd for pair-housed calves (*p* = 0.067), with 30.4% of individually housed and 24.4% of pair-housed calves leaving through either voluntary or involuntary culling between weaning and the end of first lactation. The underlying reason for this is unclear from the data collected due to there being no association found with disease occurrence or growth. However, cull levels across all age boundaries remained higher in individually housed calves. There may be underlying behavioural differences associated with the pre-weaning housing group, such that individually housed calves have a lower social rank when regrouped after weaning [48] and higher fear responses [41]. This could negatively impact the ability of individually housed calves to thrive when grouped. We showed here that individually housed heifers suffered from numerically more injuries, which may be linked to heightened fear responses when being handled or when exposed to novel situations. Future work should consider this possibility in the study design.

The overall cull level (voluntary and involuntary) pre-insemination was 2.9%, which is in agreement with that reported by [5]. However cull levels post-insemination but pre-calving were much higher at 13.0% compared with 4.2% from the same study [5]. This primarily resulted from poor fertility, as reported by others [47], with heifers failing to establish or maintain a pregnancy being voluntarily culled. The odds of heifers achieving pregnancy and reaching parturition was inversely linked to the number of inseminations the heifers received, suggesting an underlying biological reason that consistently prevented successful conception, particularly in heifers that had been individually housed. Potential causes of poor fertility were not investigated further, but could have included freemartinism, infectious diseases, metabolic or genetic causes, or lameness [49]. There may have been some effects of this farm using a commercial company that only visited the farm once per day to carry out inseminations, given that the timing of inseminations relative to the onset of oestrus and ovulation, especially when using sexed semen, is critical. Given that the mean cost of rearing a replacement heifer to the point of calving is approximately GBP 1819 [38], the potential level of farm savings if pair housing had been used for all calves is approximately GBP 12,733 per 100 heifer calves born (due to a difference in exits of 22.6% for pair-housed vs. 15.6% for individually housed calves up to the point of calving).

### 4.2. Growth Rates

The mean DLWG for heifers was not linear across the observation period [50] and was similar to that reported by Gibson et al. [51]. The slowing of DLWG as the heifers age is to be expected, with the closure of bone growth plates initiated in late puberty by high oestrogen levels leading to reduced levels of growth [52]. There was a significant association with birthweight, such that heavier birthweight calves had an increased DLWG between weaning and conception. This trend has been noted by others in pre-weaning calves [13,16,52], with this study demonstrating that the trend continues following weaning. This may be linked to physically larger calves having an increased ability to compete for food and to ingest larger meal sizes than physically smaller calves. This may suggest that housing calves in groups of a similar physical size may help to maintain consistent growth rates across whole groups of animals. We also found that heifers that experienced disease as a pre-weaning animal grew on average 40 g/day less up until conception compared with those that had been healthy, but the growth rate was not impacted by the occurrence of disease following weaning. This is supported by Donovan et al. [53], who found that the health status in the first few months of life can have significant impacts on growth, but that disease occurrence after six months of age did not impact weight gains as significantly. There was a trend for a difference in the DLWG between pre-weaning housing groups, such that individually housed calves grew 20 g/day more than pair-housed calves in the post-weaning period. This is in contrast to findings from other studies that demonstrated that pair housing can increase growth [1,2,54], but this effect does not appear to continue longer-term. The season of birth did impact the DLWG, with calves born in the winter period growing on average 50–80 g/day less than those born in other seasons. This is likely due to calves housed at lower environmental temperatures having to utilise more energy to keep warm. Work by Reuscher et al. [55] found that pair housing can help alleviate the cold stress experienced by calves and potentially have a positive impact on growth rates, although that work was conducted in outdoor hutches, which experienced lower temperatures than the sheds used in this study. Seasonality was also linked to the firstborn calf’s vitality, although this demonstrated the opposite trend to the DLWG in that calves born in the spring and summer had higher odds of experiencing disease than those born in the winter. Calves experiencing heat stress may have raised cortisol levels which can negatively impact the immune response [56], leading to increased risks of lung consolidation and respiratory disease [57,58].

### 4.3. Fertility

The mean age at conception in this study was 23.3 months, which is within the target suggested to achieve calving by the economically optimal 24 months of age [59]. However, the weight at first service demonstrated a large range (330–524 kg), even though the reported farm protocol was to start inseminating heifers at approximately 400 kg. There was an association of increasing bodyweight and age with the 305-day standardised milk yield, such that older, heavier heifers produced more milk. Others have also demonstrated this [60,61,62,63], so if the farm had ensured that heifers were not served until the target 400 kg bodyweight was achieved (nearly 80 heifers in this study), the overall milk production from the primiparous cows would have been higher, which would have increased the speed at which heifer rearing costs were recuperated. This highlights the importance of assessing ranges as well as averages when analysing farm key performance indicators (KPIs).

### 4.4. Disease

Pre-weaning calf health did not influence milk production, which is in agreement with other work [23]. However it should be noted that only weekly scoring was carried out during the pre-weaning period, which may have led to reduced sensitivity for disease detection due to some diseases being missed [13,64]. The overall binary disease occurrence was 65.3% from weaning to the end of first lactation, with 38.8% of these heifers experiencing multiple different disease events. This disease prevalence was high, with Persson Waller [65] reporting an annual mean incidence rate per 100 cow-days of veterinary-treated diseases in heifers of only 22.7 (SD 14.8). Although a high disease level was not associated with any of the measured variables, the heifers went through many changes in groups, facilities and diets during their rearing process. This may have increased their stress levels, which have been linked to negative effects on the immune system [66]. In this study, farm staff were responsible for the daily recording of diseases, and although SOP training was provided by the farm’s veterinary practice, there may have been variations between staff members and the potential for missed disease occurrences. The most prevalent problem was lameness, with this primarily involving interdigital necrobacillosis in the pre-calving period and sole haemorrhage in the post-calving period. Individually housed calves had a trend towards more lameness than pair-housed calves, but the reason for this is unclear.

### 4.5. Udder Health

With regard to udder health, heifers that were pair housed as calves had significantly increased odds of developing udder health issues as a primiparous cow, with over double the occurrence of mastitis and higher somatic cell counts compared with individually housed calves. This could influence calf housing decisions, with producers having to decide if they prioritise calf health and welfare or milk production. One possible reason for the association with pair housing is the ability of calves to cross-suck in the udder region, with this behaviour known to have occurred in the calves followed in this study—see [13] for a full analysis of the short-term impacts of paired compared to individual housing of calves. There are mixed reports of how cross-sucking can impact udder health, with Vaughan et al. (2016) [67] demonstrating that cows who were cross-sucked as heifers were not more likely to develop mastitis or have a higher somatic cell count in their first lactation. Cross-sucking is known to be a behavioural precursor to the occurrence of intersucking [35]. In this study, continued intersucking as an older heifer was only seen in a small number of animals, although this may have been underestimated as no specific monitoring other than staff observations were used to identify this behaviour. Other studies have found that intersucking is a risk factor for mastitis in heifers and can cause damage at the quarter level [28,68], which may explain the increased proportion of culled quarters in the pair-housed heifers (4.1% from individual and 6.6% from pair housing). Nutrition in the pre-weaning period of the recruited heifers in this study consisted of restricted milk feeding (6 L per day), which may have negatively impacted the occurrence of non-nutritive oral behaviours such as cross-sucking due to a lack of satiety. The impact of increased milk feeding on the occurrence of cross-sucking and the effect that this has on udder health should be studied further. It should also be noted that there are many other factors that might have influenced the development of udder health issues, such as fly control, which was not measured in this study. Despite the udder health issues, the 305-day milk yields were not associated with the housing group, and the total milk produced per calf recruited into the original study was still greater for pair-housed compared with individually housed calves (7194 kg vs. 8038 kg). This is likely due to the significantly higher hazard of exiting the herd that is linked to individual housing.

## 5. Conclusions

This study aimed to assess the longer-term impacts of individual and pair housing of pre-weaning calves on a commercial UK farm. Key findings include a reduced hazard of exiting the herd when calves were pair housed as pre-weaning calves, with potential farm level savings of GBP 12,733 per 100 heifer calves born if pair housing had been used for all calves. There was a trend for increased growth rates in individually housed calves, along with those born in the winter also having reduced growth, but seasonality was not associated with any other variables assessed. There were no negative associations of pair housing on fertility, disease (excluding udder health) or milk yields. There was, however, a significantly higher prevalence of udder health problems in heifers that had been pair housed, with over double the occurrence of mastitis and high somatic cell counts compared with individually housed calves. Even so, the total milk produced per calf recruited into the original study was still greater for pair-housed compared to individually housed calves due to the significantly higher hazard of exiting the herd that is linked to individual housing.

## Figures and Tables

**Table 1:** Summary of the total mixed ration (TMR) components fed to heifers of different ages throughout the study. The actual intakes were not measured.

	Birth to Weaning	Weaning to 10 Months	10 to 15 Months	16 to 22 Months	Lactating Primiparous Cow Ration
Crude protein, %	15.8	15.8	15.0	11.5	17.5
Starch, %	13.9	13.9	15.6	7.7	24.8
Expected intake, kg/day (dry matter intake, kg/day)	Ad libitum	11.3 (6.9)	19.2 (7.3)	34.3 (9.5)	68.0 (27.3)
Ingredients	Grass silage, chopped wheat straw, caustic wheat, mineral mix and rapeseed meal	Grass silage, chopped wheat straw, caustic wheat, mineral mix and rapeseed meal	Grass silage, chopped wheat straw, caustic wheat, waste bread, 18% heifer rearer nut, mineral mix and rapeseed meal	Grass silage, chopped wheat straw, caustic wheat, waste bread, 18% heifer rearer nut, mineral mix and rapeseed meal	Grass and maize silage, waste bread, caustic wheat and a mineral mix

**Table 2:** Summary of disease prevalence within heifers from weaning to the end of first lactation. Heifers could experience more than one disease type, so the cumulative percentage is greater than 100%.

Disease/Condition	Total (%)	Individually Housed (%)	Pair-Housed (%)
Lameness	104/418 (24.9)	44/148 (29.7) ^#^	60/270 (22.2) ^#^
Clinical mastitis	83/418 (19.9)	18/148 (12.1) *	65/270 (24.1) *
Respiratory disease	79/418 (18.9)	24/148 (16.2)	53/270 (19.6)
Miscellaneous ^1^	16/418 (3.8)	2/148 (1.4)	20/270 (7.4)
High SCC ^2^	25/418 (6.0)	6/148 (4.1)	19/270 (7.0)
Injury	26/418 (6.2)	11/148 (7.4)	15/270 (5.6)
Diarrhoea	7/418 (1.7)	3/148 (1.8)	4/270 (1.5)

^#^ indicates a trend for a Chi-squared value at *p* < 0.10; * indicates significance at *p* < 0.01. ^1^ Miscellaneous conditions include being identified as having TB through the single intradermal cervical comparator test (SICCT), hardwire disease, pyometra or an unspecific poor condition that required medical treatment. ^2^ Cattle classified as having a high somatic cell count (SCC) did not experience a case of clinical mastitis within the lactation period.

**Table 3:** Summary of the heifer signalments around the first insemination for individually housed compared to pair -housed heifers.

	Individually Housed	Pair-Housed
Number of animals	142	264
Mean age at first insemination (SD), days	396 (12.5)	399 (27.8)
Mean number of inseminations (SD, range)	2.5 (1.7, 1–7)	2.5 (1.5, 1–7)
Mean DLWG until pregnancy (SD), kg/day	0.73 (0.13)	0.72 (0.12)
Mean bodyweight at first insemination (SD, range), kg	417 (34.8, 340–524)	417 (31.4, 330–500)
Mean age at first calving (SD), days	698.2 (34.0)	697.8 (35.1)

## Data Availability

The data presented in this study are available on request from the corresponding author.

## Appendix A

**Table A1:** Summary of the statistical models used in this study, showing the dependent and independent variables. ‘y’ indicates yes for inclusion of the variable within the model detailed per row.

Model Type	Dependent Variables	Pen	Housing Group	Season of Birth	Birthweight	DLWG	Disease Pre-Weaning	Disease Post-Weaning	Disease (Exc. Udder Health) Post-Weaning	Disease Pre-Insemination	Age at First Insemination	Number of Inseminations	Bodyweight at First Insemination	Age at Calving
Cox regression	Survival		y				y	y						
Generalised estimating equations	Disease (exc. udder health) from weaning to end of first lactation	y	y	y		y	y							
	Udder health disease from weaning to end of first lactation	y	y	y		y	y		y					y
	Successful pregnancy and parturition	y	y	y		y	y			y	y	y		
	Mortality rate of firstborn calves	y	y	y		y				y		y		y
	Disease occurrence in firstborn calves	y	y	y		y				y		y		y
Linear mixed model	DLWG from birth to confirmation of pregancy	y	y	y	y		y			y				
	Number of inseminations given	y	y	y		y	y			y	y		y	
	Age at calving	y	y	y		y	y			y			y	
	Energy-corrected 305 d milk yield	y	y	y		y	y	y					y	y
